# Alterations in the Fatty Acid Composition in Infant Formulas and ω3-PUFA Enriched UHT Milk during Storage

**DOI:** 10.3390/foods8050163

**Published:** 2019-05-13

**Authors:** Luis Miguel Rodríguez-Alcalá, María V. Calvo, Javier Fontecha, Leocadio Alonso

**Affiliations:** 1 Centro de Biotecnología e Química Fina, Universidade Católica Portuguesa (UCP), 4200-374 Porto, Portugal; lalcala@porto.ucp.pt; 2 Instituto de Investigación en Ciencias de la Alimentación (CSIC-UAM), 28049 Madrid, Spain; mv.calvo@csic.es (M.V.C.); j.fontecha@csic.es (J.F.); 3 Instituto de Productos Lácteos de Asturias (CSIC), 33300 Asturias, Spain

**Keywords:** infant formulas, ω3-PUFA, *trans* fatty acids, UHT milk, storage

## Abstract

ω3-polyunsaturated fatty acids (PUFA) are known to have several beneficial effects, such as preventing the occurrence of cardiovascular events in adults and improving the development of the central nervous system during fetal life and childhood. Dairy products enriched in PUFA are now available on the market and can help consumers to meet the ω3-PUFA daily intake recommendations. Although PUFA are prone to oxidation, little information exists regarding the alterations they undergo when products containing PUFA are stored. In this study, five infant formulae (IF) and five ω3-PUFA enriched Ultra High Temperature (UHT)-milk products were examined during storage at room temperature in terms of fatty acid (FA) composition and *trans* fatty acid (tFA) content until the end of shelf life by chromatography techniques. The IF included two follow-on formulae, two first-age formulae and a special medical purpose formula with different fat contents (from 2.6% to 27.7%). In the ω3-PUFA enriched UHT-milk products the milk fat was replaced by eicosapentanoic fatty acid (EPA) and docosahexaenoic acid (DHA) rich oils. The fatty acid content of all samples remained stable whereas some variations were observed for the tFA isomer content (*p* < 0.05) in the UHT-milk samples.

## 1. Introduction

The consumption of whole milk has declined in recent years, partly due to consumer perceptions concerning health and welfare. In the European Union (EU), the costs associated with cardiovascular disease (CVD) increased from €192 billion in 2009 to a current €210 billion per year. Every year CVD causes 3.9 million deaths in Europe and over 1.8 million in the EU [1]. The consumption of fish rich in ω3-polyunsaturated fatty acids (PUFA) has been associated with a reduced risk of CVD, both in terms of incidence and mortality [2,3], and these days there are many skimmed and semi-skimmed milk and infant formulas (IF) available that are enriched in ω3-PUFAs [4,5,6]. The consumption of some of these products has been shown to reduce the plasma level of triacylglycerol, total cholesterol and Low Density Lipoprotein (LDL) cholesterol levels [7], to help protect against certain types of cancer [8], to help maintain neurological function [9], to aid in the prevention of allergic diseases [10] and to reduce the risk of several CVD, including in patients with metabolic syndrome [11]. ω3-PUFA is also important for the proper development of the central nervous system during fetal life, childhood and adults [12,13]. Arachidonic acid (AA) is an important structural fatty acid in the brain, while docosahexanoic acid (DHA) is important in the synthesis of the membrane phospholipids needed for neurite elongation and it has also been suggested ω3-PUFA may play a role in brain health during aging [14].

Given the susceptibility of PUFA to oxidation, fortified food products need to be processed, packed, stored and distributed under conditions that avoid this problem, i.e., low levels of oxygen, ultraviolet (UV) light, low temperature and humidity, and low product concentrations of metal ions (especially Fe, Cu and Mn). The most suitable foods for fortification with ω3-PUFA are therefore those that are frequently consumed and stored for only short periods at low temperature in airtight, light-excluding packages [15,16,17]. Good examples of such foods are dairy products. UHT milk is the most consumed type of milk in some European countries, and UHT treatments have been shown effective in maintaining the characteristics and nutritional value of milk unaltered for long periods of time at room temperature. IF also contain PUFA, provided by a mixture of vegetable and fish oils. These formulas can ensure that infants who are undernourished or premature have an adequate supply of these fatty acids [18]. They must, therefore, also be stored under similar conditions. Both types of product also contain *trans* monoenoic isomers of fatty acids (tFA) which might also suffer oxidation damage over long storage times.

The aim of the present work was to study the changes in the fatty acid (FA) composition and tFA content of IF and ω3-PUFA enriched UHT milk and at room temperature during storage until the end of shelf life by gas chromatographic (GC) analysis using a flame ionization detector (FID).

## 2. Materials and Methods

### 2.1. Samples and Reagents

Five IF products (covering three brands) were examined. IF1 was for 1–3 year-olds (2.6% fat); IF2 a starter formula (27.7% fat); IF3 a follow-up formula for babies with minor digestive problems (20% fat); IF4 for premature babies (26% fat), and IF5 a follow-up IF (5.24%) dehydratated. Their manufacturer declared shelf life of IF1 was 12 weeks; that of all others was 48 weeks.

Five ω3-PUFA enriched UHT-milk products (also covering three brands) were examined (Mw1, Mw2, Mw3, Mw4, and Mw5). The Mw type samples were all UHT milk based, in which the milk fat had been replaced by high eicosapentanoic fatty acid (EPA) and docosahexaenoic acid (DHA) content oils. Mw4 was also fortified with folic acid (80 mg/100 mL). All samples had a manufacturer declared shelf life of 12 weeks.

All samples examined were obtained from local supermarkets. All analyses were performed in triplicate (using sample replicates from the same manufacturing lots as far as possible to avoid variation due to processing). All samples were stored in their packages without opening, in the dark, at room temperature.

Methyl esters of ω3-PUFA and CLA standards purchased from Nu-Check Prep. Inc. (Elysian, MN, USA). Saturated and methyl *trans* monoenoic fatty acid methyl esters standards purchased from, (Sigma Chemical, St. Louis, MO, USA). Anhydrous milk fat with a certified fatty acid composition (reference material BCR-164, obtained from the Commission of the European Communities, Brussels, Belgium). Thin layer chromatography (TLC) glass plates (20 × 20 cm) with silica gel (0.25 mm) (Merck, Darmstadt, Germany); Silver nitrate, 2′,7′-dichlorofluorescein, potassium hydroxide and solvents purchased from (Panreac, Barcelona, Spain).

### 2.2. Fatty Acids Analysis

Analysis of the PUFA and tFA contents were made upon reception of the products by the selling supermarkets (i.e., close to the time of production), and at week 6 of storage for IF1, and at week 24 and 48 for the rest of the IF products. For the UHT-milk products, analyses were made at the reception of the samples, in the middle of the shelf life period, and at expiry.

### 2.3. Lipid Extraction

Lipid extraction was performed according to [19] and stored at −20 °C until analysis.

### 2.4. Preparation of Fatty Acid Methyl Esters

For preparation of fatty acid methyl esters (FAME) of milk fat, 0.1 g of fat was dissolved in 1 mL of hexane, and 0.05 mL of 2 N potassium hydroxide in methanol was added as described [20].

### 2.5. Fractioning by AgNO_3_-TLC of FAME

FAME were fractionated according to the number and geometry of double bonds by AgNO_3_-TLC following the procedure [21]. After the chromatographic run, the bands corresponding to the saturated and *trans* monoenoic FAME were scraped, dissolved in heptane and used for GC analysis. To calculate the total content of *trans*-C18:1 isomers, the ratio C18:0 to total *trans* C18:1 was determined in the saturated plus *trans* monoenoic C18:1 AgNO_3_-TLC fraction and was related to the C18:0 content of total FAME. To calculate the total content of *trans* C16:1 isomers, the ratio C18:0 to total *trans* C16:1 determined in the saturated plus *trans* monoenoic C16:1 AgNO_3_-TLC fraction by GC was related to the C18:0 content of total FAME.

### 2.6. GC Analysis of Total and Trans Fatty Acids

FAME solution (0.2 μL) was injected into an Autosystem model GC (Perkin-Elmer Co., Beaconsfield, UK) equipped with a flame ionization detector (FID). Analysis were performed with a CP Sil 88 column (100 m × 0.25 mm i.d.) containing 100% cyanopropyl siloxane as the stationary phase with a film thickness 0.20 μm (Chrompack, Middelburg, The Netherlands) [21]. The initial temperature of 70 °C was maintained for 3 min, then raised to 175 °C at a rate of 9 °C/min, maintained for 27.5 min, and then increased to 210 °C at a rate of 1.3 °C/min for 10 min. The split ratio was 1:50, and hydrogen was the carrier gas with a head pressure of 1.2 kg/m^2^. The injector and detector temperatures were 250 °C. For quantitative determinations of FAME of AgNO_3_-TLC fractions, the column and chromatographic conditions were the same as total FAME. Duplicate analyses were performed for each sample.

### 2.7. Statistical Analysis

Differences in the FA compositions and tFA contents of the analyzed products occuring over time were analysed by ANOVA plus Bonferroni correction, with storage as a factor. Significance was set at *p* < 0.05. All calculations were performed using the SPSS package for Windows v.13 (SPSS, Chicago, IL, USA).

## 3. Results and Discussion

### 3.1. Infant Formulas

**Figure 1:**
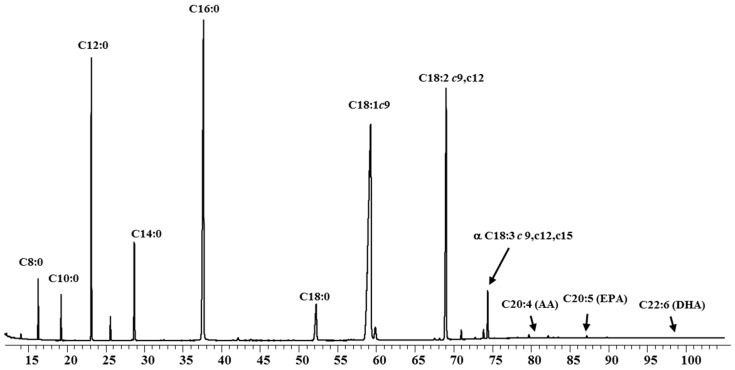
Fatty acid profile of infant formula (IF2) by gas chromatographic. c: *cis*; t: *trans*; AA: arachidonic acid; EPA: eicosapentanoic fatty acid; DHA: docosahexanoic fatty acid.

Table 1 shows the FA composition (g FA/100 g of total FA) of the IF products at reception and at the end of their shelf lives (since no significant changes occurred, the mid storage values are not shown). Few studies related to the composition of FA in IF during storage are available in the literature. All the samples, except IF4, the mean values for saturated fatty acids (SFA) 39.68–62.40% were slightly higher than those found in human milk (30–50%) [22]. All the IF except IF4 had palmitic acid (C16:0) 16.58–22.78% as the main FA. For IF4 the most important fatty acids were caprilic (C8:0) 31.90% and capric (C10:0) 20.67%. In IF2 (Figure 1) and IF3 were found contents of lauric acid (C12:0) above 10% and for IF1 and IF5, stearic acid (C18:0) had levels of 6.96% and 7.72% respectively. Sample IF4 showed high concentration of the medium chain fatty acids (C8:0, C10:0). As this IF is designed for premature babies, that compounds are suitable for their digestibility, as energy source [23]. In all samples, oleic acid (C18:1) showed 22.85–38.76% in agreement with the recommended contents 24–40% to these milks [24,25]. The composition of IF5 samples had a total content in PUFA of 30.47% due to its concentrations in linoleic acid (LA) 26.65%. The recommend ω6/ω3 ratio given for several guidelines is 5–10:1 [22] being, in the assayed samples, from 12 to 6. In general, DHA was not detected except for IF4, in concentrations lower than the normal content in human milk 0.3%.

It has been reported by other authors, studies of the evolution of the FA and volatile profiles in commercial IF, storage at room temperature until 12–18 months after opening, losses in the contents of LA. Although concentrations of propanal and hexanal increased during the assayed time [26], there was no correlation with LA and linoleic acid (LnA). Also, there were no alterations in the content of other PUFA like DHA and AA. On the other hand, in IF enriched with PUFA and stored 18 months at 25 °C and 40 °C, was reported increments in the peroxide value and volatile compounds during the conservation period, but only slightly losses in LA, showing an acceptable stability of the lipid fraction [27,28,29]. The absence of patterns in the behavior of oxidation compounds, fatty acids and volatile moieties in IF in mentioned works, as well in the fatty acid composition in the present work, support the statement that the lipid fraction of IF is not altered during storage.

In samples IF2, IF3 and IF4 were not detected tFA. The concentration in IF1 was 0.26% and 0.98% in IF5. According to the distribution of the individual isomers of *trans* C18:1 expressed as (mg FA/100 g of total FA) (Table 2) showed that *trans* 11 C18:1 (vaccenic acid, VA) and *trans* 13–14 C18:1 were the main compounds, but the concentration was higher in samples IF5 than IF1 308.81 mg/100 g total FA–30.64 mg/100 g total FA. This was also observed for total *trans* C16:1, *trans* 6–8 C18:1, *trans* 9 C18:1 (elaidic acid, EA) and *trans* 10 C18:1. *Trans* 4 C18:1 was not detected in IF1.

Previous research works performed in commercial IF have shown that the total content and distribution of tFA can vary in the range of 0.2–2% according to the composition of the lipids sources agree with that found in samples IF1 [30]. However, other investigations also in commercial and experimental IF, found that the contents of these moieties were under 0.1% or not detected [27,30,31]. The distributions of tFA for IF1 in the present work, agree with that reported in [4,32] for the same kind of samples. On the other hand, IF5 showed the higher amounts of VA and the presence of SFA that points out to the presence of milk fat in this product.

Previous studies have shown that IF are susceptible to undergo oxidation reactions after their processing and conservation time, due to the high contents in PUFA and manufacturers recommend consumption of the product in a month [33]. Lipid oxidation reactions occur by way of an interaction with oxygen or oxidized amino and sulphur groups, they are also affected by temperature and a permeability to oxygen/light when packaged. Few works are focused specifically in the alterations of the lipid profile along storage. In previous studies carried out in IF during four years of storage at ambient temperature and blanketed with inert atmosphere, were found lower concentrations of oleic and LnA after the first year of storage while this was observed for LA in the third year [4,33]. Nevertheless, no alterations in the contents of tFA were observed, supporting the results found in this study.

### 3.2. ω3-PUFA-Enriched UHT-Milk Products

**Figure 2:**
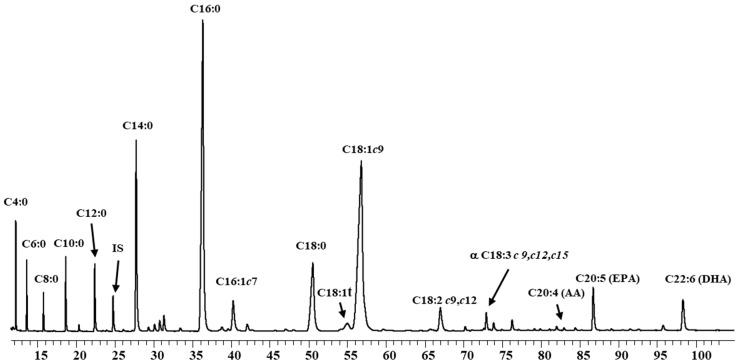
Fatty acid profile of PUFA UHT-enriched milk (Mw2) by gas chromatographic. c: *cis*; t: *trans*; AA: arachidonic acid; EPA: eicosapentanoic fatty acid; DHA: docosahexanoic fatty acid; IS: Internal Standard.

Table 3 shows the FA composition (g FA/100 g of total FA) of the ω3-PUFA enriched UHT-milk products at reception and at the end of their shelf lives (since no significant changes occurred, the mid storage values are not shown). The contents of PUFA ranged from 9.89% in Mw2 (Figure 2) to 15.58% in Mw4 samples. The main FA of this group was LA whose highest percentage was registered for the Mw4 samples 14.12%. EPA ranged from 2.47% in the Mw2 samples to 0.19% in Mw4 samples. High DHA concentrations were found in the Mw2 samples. The EPA+DHA concentrations in Mw1 and Mw4 are in accordance with the stated values by the manufacturer while for Mw2, Mw3 and Mw4 were slightly lower. As mentioned above, the ω6/ω3 ratio has been recommended to be 5–10:1. Samples Mw1 and Mw5 are in agreement with these values but in the rest of the samples were higher as well as the LA/LnA ratio. MUFA represented the main fraction, due to the contents of C18:1, reaching a 76.22% in the Mw4 samples. The amounts of SFA varied from 48.67% in Mw2 to 11.17% in Mw. The most abundant saturated compounds were C16:0 22.71% for Mw2 followed by C18:0 8.19% for Mw3. Mw2 and Mw3 also have important amounts of myristic acid C14:0 7.27–2.20%.

In the industrialized countries, DHA + EPA intake is below 100 mg/person/day, while LnA consumption is 0.7–2.3 g/person/day and 7–19 g/person/day for LA [3]. The European Food Safety Authority recommends a consumption of EPA+DHA of 250 mg/person/day, 2 g for LnA and 6 g for LA [34]. The results of the present study show that a serving size (250 mL) of each assayed product provides 107.5 mg/person/day (Mw1), 190 mg/person/day (Mw2) and 67.5 mg/person/day (Mw3) and a range of 9–2 of mg/person/day LnA. A 250 mL/day intake of these products alone does not cover the recommended guidelines. Furthermore, none of these dairy is an important source of LnA.

The results obtained in this work do not show changes (*p* < 0.05) in SFA, MUFA and PUFA of dairy products enriched with ω3-PUFA during the time of conservation. Although UHT processing is used to assure the microbiological quality of foods, exists diverse lipases highly thermoresistant, not inactivated after treatment and which have as substrate specific fatty acids (C10:0, C12:0, C14:0 and C18:0) [35,36], in our study we did not find any alterations in the short and medium chain fatty acids for the treatment and storage. The contents and distribution of the tFA (mg FA/100 g of total FA) are shown in Table 4. According to the distribution of the tFA, high concentrations of VA, *trans* 13–14 C18:1 and *trans* 10 C18:1 acids were found in Mw2. EA was the third most important tFA in the Mw2 and Mw4 samples while in Mw3 and Mw5 was *trans* 12 C18:1. Regarding the content of tFA, there was a significant difference (*p* < 0.05) for the *trans* 6–8 (19.95 mg FA/100 g of total FA vs 16.39 mg FA/100 g of total FA and trans 10 (32.28 mg FA/100 g of total FA vs 36.19 mg FA/100 g of total FA for the sample Mw1 and for the trans 6–8 (19.9 mg FA/100 g of total FA vs 14.19 mg FA/100 g of total FA for Mw5 and EA (25.29 mg FA/100 g of total FA vs 19.07 mg FA/100 g of total FA for the sample Mw5. In the present work, the ω3-PUFA enriched commercial samples treated by UHT, according to the existing bibliography leads, to an environment of reduced potential redox and in where the oxygen content is low [37]. In addition, considering the fact that the experience was made in the dark and without opening the samples, explains the absence of variation in the FA profile during the conservation.

## 4. Conclusions

The results of this investigation disclosed that during storage at room temperature until their expiry date, the fatty acid profile (SFA, MUFA and PUFA) of IF and ω3-PUFA enriched milk samples was stable, except for the content of tFA. There was a significant difference (*p* < 0.05) for the *trans* 6–8 C18:1 and *trans* 10 C18:1 for the sample Mw1 and for the *trans* 6–8 C18:1 and *trans* 10 C18:1 for the sample Mw5 of the ω3-PUFA enriched milk.

## Figures and Tables

**Table 1:** Fatty acid composition (FA) (g FA/100 g of total FA ± Standard Deviation (SD) at reception (R) and at the end of the shelf life (SL) in commercial infant formulas.

FA	IF1		IF2		IF3		IF4		IF5	
	R	SL	R	SL	R	SL	R	SL	R	SL
C4:0	2.34 ± 0.25	2.27 ± 0.28	0.05 ± 0.01	0.06 ± 0.01	0.08 ± 0.01	0.07 ± 0.01	0.08 ± 0.01	0.09 ± 0.02	2.22 ± 0.21	2.14 ± 0.24
C6:0	1.54 ± 0.08	1.48 ± 0.12	0.22 ± 0.04	0.24 ± 0.05	0.15 ± 0.04	0.17 ± 0.04	0.30 ± 0.08	0.32 ± 0.07	1.25 ± 0.15	1.19 ± 0.12
C8:0	0.82 ± 0.05	0.85 ± 0.08	2.36 ± 0.32	2.46 ± 0.21	1.55 ± 0.05	1.53 ± 0.12	32.62 ± 2.65	31.90 ± 3.34	0.80 ± 0.08	0.73 ± 0.08
C10:0	0.96 ± 0.06	1.89 ± 1.15	1.74 ± 0.19	1.69 ± 0.09	1.28 ± 0.09	1.21 ± 0.10	20.05 ± 1.96	20.67 ± 1.56	1.55 ± 0.10	1.65 ± 0.14
C10:1	0.24 ± 0.02	0.22 ± 0.05	0.02 ± 0.00	0.02 ± 0.00	0.01 ± 0.00	0.01 ± 0.00	0.03 ± 0.01	0.03 ± 0.01	0.12 ± 0.03	0.10 ± 0.02
C12:0	2.22 ± 0.19	2.17 ± 0.26	10.86 ± 1.19	10.75 ± 1.20	13.54 ± 1.58	12.47 ± 1.63	0.32 ± 0.07	0.35 ± 0.06	1.92 ± 00.12	1.87 ± 0.11
C14:0	6.80 ± 0.39	6.75 ± 0.98	3.95 ± 0.36	3.80 ± 0.15	4.52 ± 0.61	4.63 ± 0.29	0.49 ± 0.09	0.42 ± 0.07	6.06 ± 0.68	5.86 ± 0.31
C14:1	0.51 ± 0.09	0.55 ± 0.08	0.01 ± 0.00	0.01 ± 0.00	0.01 ± 0.00	0.01 ± 0.00	0.02 ± 0.00	0.02 ± 0.00	0.52 ± 0.09	0.54 ± 0.08
C15:0	0.75 ± 0.08	0.71 ± 0.09	0.03 ± 0.00	0.04 ± 0.01	0.05 ± 0.01	0.05 ± 0.01	0.03 ± 0.01	0.04 ± 0.01	0.55 ± 0.08	0.59 ± 0.07
C16:0	22.18 ± 1.21	21.16 ± 1.19	23.95 ± 1.95	22.78 ± 1.12	17.68 ± 2.35	16.58 ± 1.94	7.15 ± 0.68	6.84 ± 0.45	19.26 ± 2.01	18.25 ± 1.75
C16:1	0.98 ± 0.08	1.07 ± 0.09	0.13 ± 0.04	0.15 ± 0.04	0.11 ± 0.03	0.12 ± 0.05	0.07 ± 0.01	0.08 ± 0.02	0.82 ± 0.16	0.87 ± 0.07
C17:0	0.42 ± 0.05	0.45 ± 0.08	0.07 ± 0.01	0.07 ± 0.01	0.05 ± 0.01	0.05 ± 0.01	0.06 ± 0.01	0.05 ± 0.01	0.42 ± 0.08	0.39 ± 0.05
C17:1 c	0.19 ± 0.04	0.16 ± 0.04	0.04 ± 0.01	0.04 ± 0.01	0.03 ± 0.00	0.03 ± 0.01	0.03 ± 0.00	0.03 ± 0.01	0.12 ± 0.04	0.14 ± 0.04
C18:0	7.10 ± 0.51	6.96 ± 1.16	3.12 ± 0.09	2.99 ± 0.25	2.71 ± 0.08	2.61 ± 0.12	1.69 ± 0.15	1.58 ±0.06	7.75 ± 1.11	7.72 ± 1.24
C18:1 t	0.23 ± 0.06	0.26 ± 0.06	n.d	n.d	n.d	n.d	n.d	n.d	1.02 ± 0.09	0.98 ± 0.12
C18:1 c9	22.78 ± 1.65	22.64 ± 1.45	34.01 ± 2.95	35.34 ± 2.66	39.95 ± 3.67	38.76 ± 3.01	23.69 ± 2.32	22.58 ± 1.65	25.78 ± 2.45	23.57 ± 2.09
C18:1 c10	2.15 ± 0.32	2.03 ± 0.31	1.21 ± 0.08	1.17 ± 0.12	1.66 ± 0.17	1.72 ± 0.21	0.55 ± 0.09	0.62 ± 0.07	1.01 ± 0.08	0.93 ± 0.16
C18:2	1.05 ± 0.12	0.99 ± 0.08	0.16 ± 0.03	0.17 ± 0.03	0.16 ± 0.07	0.17 ± 0.08	0.06 ± 0.01	0.07 ± 0.01	0.62 ± 0.04	0.57 ± 0.07
C18:2 c9,c12	2.19 ± 0.66	19.90 ± 1.66	45.23 ± 3.65	14.52 ± 1.68	15.98 ± 1.96	15.83 ± 1.64	12.05 ± 1.31	11.11 ± 0.25	27.78 ± 2.19	26.65 ± 2.66
C20:0	0.21 ± 0.08	0.23 ± 0.05	0.31 ± 0.04	0.32 ± 0.08	0.31 ± 0.08	0.32 ± 0.05	0.19 ±0.04	0.14 ± 0.04	0.22 ± 0.06	0.19 ± 0.04
C20:1 c9	0.13 ± 0.04	0.15 ± 0.04	0.02 ± 0.00	0.02 ± 0.00	0.10 ± 0.02	0.12 ± 0.03	0.02 ± 0.00	0.02 ± 0.00	n.d	n.d
C20:1 c11	0.05 ± 0.01	0.06 ± 0.01	0.32 ± 0.06	0.37 ± 0.07	0.42 ± 0.06	0.38 ± 0.06	0.18 ± 0.03	0.22 ± 0.04	0.06 ± 0.02	0.05 ± 0.01
C18:3 n3	1.79 ± 0.10	1.63 ± 0.12	2.13 ± 0.12	2.02 ± 0.16	2.25 ± 0.16	2.17 ± 0.17	1.35 ± 0.09	1.22 ± 0.07	2.84 ± 0.26	2.73 ± 0.15
CLA c9, t11	0.65 ± 0.09	0.55 ± 0.08	0.03 ± 0.00	0.03 ± 0.00	0.02 ± 0.00	0.02 ± 0.00	0.03 ± 0.01	0.04 ± 0.01	0.59 ± 0.10	0.53 ± 0.07
C20:3 n3	0.01 ± 0.00	0.01 ± 0.01	0.06 ± 0.01	0.07 ± 0.01	0.19 ± 0.03	0.21 ± 0.06	0.02 ± 0.00	0.02 ± 0.00	n.d	n.d
C20:4 AA	0.07 ± 0.01	0.08 ± 0.02	0.03 ± 0.01	0.02 ± 0.00	0.07 ± 0.01	0.06 ± 0.01	0.05 ± 0.01	0.05 ± 0.01	n.d	n.d
EPA	0.02 ± 0.00	0.02 ± 0.00	0.09 ± 0.02	0.08 ± 0.01	0.03 ±0.00	0.03 ± 0.01	0.07 ± 0.02	0.06 ±0.02	n.d	n.d
C22:5 DPA	0.04 ± 0.01	0.04 ± 0.01	0.06 ± 0.01	0.06 ± 0.01	n.d	n.d	0.03 ± 0.01	0.03 ± 0.01	n.d	n.d
C22:6 DHA	n.d	n.d	n.d	n.d	n.d	n.d	0.22 ± 0.06	0.18 ± 0.04	n.d	n.d
SFA	45.34 ± 3.39	44.92 ± 3.79	46.66 ± 2.98	45.19 ± 3.24	42.23 ± 3.65	39.68 ± 3.14	62.98 ± 4.98	62.40 ± 4.05	42.01 ± 3.66	40.59 ± 3.18
MUFA	27.26 ± 1.66	26.88 ± 2.65	35.76 ± 3.12	37.10 ± 3.01	41.92 ± 3.58	41.15 ± 3.71	24.89 ± 2.21	23.61 ± 2.35	29.34 ± 2.57	26.20 ± 2.14
PUFA	27.40 ± 1.98	28.20 ± 2.35	17.58 ± 1.96	16.99 ± 1.68	15.85 ± 2.01	18.48 ± 1.81	12.13 ± 1.35	12.77 ± 1.34	28.65 ± 2.01	30.47 ± 2.94
n6/n3		11.86 ± 1.29		6.71 ± 0.66		7.23 ± 0.57		7.519 ± 0.46		9.77 ± 0.91

FA: fatty acid; c: *cis*; t: *trans*; AA: arachidonic acid; EPA: eicosapentanoic fatty acid; DPA: docosapentanoic fatty acids; DHA: docosahexanoic fatty acid; SFA: saturated fatty acid; MUFA: monounsaturated fatty acid; PUFA: polyunsaturated fatty acid R: reception; shelf life (SL) 12 weeks (IF1) and 48 weeks (IF2, IF3,IF4 and IF5) at room temperature; n: omega; n.d: not detected samples; *n* = 9.

**Table 2:** *Trans* monoenoic fatty acid isomers composition (mg FA/100 g of total FA ± Standard Deviation (SD) at the reception (R) and at the end of shelf life (SL) in commercial infant formula.

tFA	IF1		IF5	
	R	SL	R	SL
Total *trans* C16:1	6.92 ± 0.58	6.23 ± 0.67	66.75 ± 6.85	68.7 ± 6.14
*trans* 4 C18:1	n.d	n.d	6.02 ± 0.58	6.51 ± 5.92
*trans* 5 C18:1	5.02 ± 0.48	5.17 ± 0.42	6.81 ± 0.61	6.93 ± 0.48
*trans* 6-8 C18:1	15.12 ± 1.35	16.94 ± 1.74	13.59 ± 1.41	14.52 ± 1.32
*trans* 9 C18:1	38.75 ± 3.45	37.64 ± 3.25	43.25 ± 4.20	41.74 ± 4.06
*trans* 10 C18:1	40.02 ± 3.65	41.87 ± 4.12	63.79 ± 6.18	61.4 ± 5.58
*trans* 11 C18:1	48.25 ± 4.01	50.53 ± 5.12	989.27 ± 89.51	957.21 ± 94.78
*trans* 12 C18:1	18.08 ± 1.65	17.26 ± 1.66	112.23 ± 9.65	115.02 ± 10.20
*trans* 13-14 C18:1	29.12 ± 2.25	30.64 ± 3.20	291.64 ±18.22	308.81 ± 28.45
*trans* 15 C18:1	10.14 ± 1.14	11.12 ± 1.12	132.56 ± 12.94	140.05 ± 13.51
*trans* 16 C18:1	10.08 ± 1.05	11.03 ± 1.05	127.65 ± 12.10	138.40 ± 12.11

tFA: *trans* fatty acid; R: reception; shelf life (SL) 12 weeks (IF1) and 48 weeks (IF5) at room temperature; n.d: not detected; *n* = 9.

**Table 3:** Fatty acid composition (g FA/100 g of total FA ± Standard Deviation (SD) at the reception (R) and at the end of the shelf life (SL) in commercial PUFA UHT-enriched milk.

FA	Mw1		Mw2		Mw3		Mw4		Mw5	
	R	SL	R	SL	R	SL	R	SL	R	SL
C4:0	0.13 ± 0.03	0.12 ± 0.01	2.41 ± 1.33	2.32 ± 0.21	0.71 ± 0.06	0.67 ± 0.04	0.12 ± 0.03	0.10 ± 0.02	0.17 ± 0.04	0.15 ± 0.02
C6:0	0.08 ± 0.02	0.09 ± 0.02	1.49 ± 1.01	1.43 ± 0.10	0.48 ± 0.04	0.42 ± 0.03	0.08 ± 0.01	0.07 ± 0.02	0.12 ± 0.02	0.10 ± 0.02
C8:0	0.07 ± 0.01	0.06 ± 0.01	0.72 ± 0.08	0.78 ± 0.07	0.21 ± 0.03	0.23 ± 0.02	0.06 ± 0.01	0.04 ± 0.01	0.06 ± 0.02	0.07 ± 0.01
C10:0	0.14 ± 0.03	0.12 ± 0.03	1.75 ± 0.10	1.64 ± 0.12	0.42 ± 0.05	0.47 ±0.04	0.14 ± 0.03	0.08 ± 0.02	0.21 ± 0.3	0.12 ± 0.02
C10:1	0.01 ± 0.00	0.01 ± 0.00	0.22 ± 0.03	0.19 ± 0.05	0.05 ± 0.01	0.05 ± 0.01	0.01 ± 0.00	0.01 ± 0.00	0.01 ± 0.00	0.01 ± 0.00
C12:0	0.19 ± 0.03	0.21 ± 0.04	1.96 ± 0.12	1.85 ± 0.12	0.66 ± 0.04	0.59 ± 0.04	0.16 ± 0.02	0.14 ± 0.03	0.41* ± 0.04	0.25 ± 0.03
C14:0	1.09 ± 0.09	1.03 ± 0.10	7.95 ± 0.53	7.27 ± 0.71	2.31 ± 0.21	2.20 ± 0.15	0.58 ± 0.06	0.49 ± 0.04	1.09 ± 0.11	1.01 ± 0.03
C15i	0.02 ± 0.00	0.02 ± 0.00	0.17 ± 0.03	0.20 ± 0.03	0.07 ± 0.01	0.08 ± 0.02	0.01 ± 0.00	0.01 ± 0.00	0.02 ± 0.00	0.02 ± 0.00
C15ai	0.02 ± 0.00	0.02 ± 0.00	0.28 ± 0.02	0.32 ± 0.02	0.08 ± 0.02	0.10 ± 0.01	0.02 ± 0.00	0.02 ± 0.00	0.02 ± 0.00	0.03 ± 0.01
C14:1	0.04 ± 0.01	0.03 ± 0.01	0.59 ± 0.06	0.52 ± 0.05	0.19 ± 0.03	0.15 ± 0.02	0.02 ± 0.00	0.02 ± 0.00	0.03 ± 0.01	0.03 ± 0.01
C15:0	0.08 ± 0.02	0.09 ± 0.02	0.62 ± 0.05	0.74 ± 0.06	0.20 ± 0.02	0.24 ± 0.02	0.05 ± 0.01	0.06 ± 0.02	0.07 ± 0.02	0.08 ± 0.02
C16i	0.01 ± 0.00	0.01 ± 0.00	0.23 ± 0.03	0.19 ± 0.03	0.04 ± 0.00	0.05 ± 0.01	0.01 ± 0.00	0.01 ± 0.00	0.01 ± 0.00	0.01 ± 0.00
C16:0	9.05 ± 1.32	8.68 ± 0.08	23.69 ± 1.45	22.71 ± 2.01	12.29 ± 0.11	12.14 ± 1.01	9.19 ± 0.08	8.07 ± 0.08	6.18 ± 0.78	5.82 ± 0.08
C16:1t	0.02 ± 0.00	0.02 ± 0.00	0.04 ± 0.01	0.05 ± 0.01	0.06 ± 0.01	0.07 ± 0.02	0.02 ± 0.00	0.03 ± 0.00	0.03 ± 0.01	0.04 ± 0.01
C16:1c	0.65 ± 0.10	0.72 ± 0.05	2.36 ± 0.16	2.27 ± 0.25	0.66 ± 0.04	0.57 ± 0.04	0.16 ± 0.02	0.18 ± 0.02	0.79 ± 0.06	0.74 ± 0.05
C17:0	0.11 ± 0.03	0.09 ± 0.02	0.48 ± 0.07	0.52 ± 0.06	0.15 ± 0.02	0.18 ± 0.02	0.09 ± 0.01	0.08 ± 0.02	0.06 ± 0.01	0.07 ± 0.01
C17:1	0.08 ± 0.02	0.07 ± 0.02	0.11 ± 0.03	0.14 ± 0.02	0.07 ± 0.02	0.07 ± 0.01	0.03 ± 0.00	0.03 ± 0.00	0.06 ± 0.01	0.07 ± 0.02
C18:0	6.05 ± 0.71	5.71 ± 0.06	7.12 ± 0.30	7.93 ± 0.54	8.19 ± 0.07	7.97 ± 0.06	6.71 ± 0. 61	6.67 ± 0.06	3.21 ± 0.31	3.13 ± 0.05
C18:1 t	0.25 ± 0.04	0.27 ± 0.03	1.71 ± 0.09	1.80 ± 0.21	0.31 ± 0.04	0.25 ± 0.03	0.51 ± 0.06	0.43 ± 0.05	0.32 ± 0.04	0.40 ± 0.03
C18:1 c9	66.68 ± 4.32	65.35 ± 4.52	31.05 ± 1.69	30.16 ± 3.15	59.15 ± 4.01	58.32 ± 5.21	66.09 ± 5.01	64.80 ± 5.14	71.85 ± 6.08	70.02 ±6.12
C18:1 11c	1.65 ± 0.35	1.57 ± 0.11	1.72 ± 0.16	1.89 ± 0.25	1.61 ± 0.18	1.56 ± 0.14	1.78 ± 0.25	1.64 ± 0.17	1.96 ± 0.17	1.83 ± 0.22
C18:1 12c	0.12 ± 0.03	0.14 ± 0.03	0.19 ± 0.08	0.22 ± 0.03	0.12 ± 0.04	0.13 ± 0.04	0.12 ± 0.04	0.18 ± 0.03	0.13 ± 0.04	0.15 ± 0.04
C18:1 13c	0.02 ± 0.00	0.02 ± 0.00	0.05 ± 0.01	0.05 ± 0.01	0.02 ± 0.00	0.02 ± 0.00	0.03 ± 0.01	0.03 ± 0.01	0.02 ± 0.00	0.02 ± 0.00
C18:2 c9,t13	0.31 ± 0.04	0.27 ±0.03	0.10 ± 0.02	0.11 ± 0.02	0.26 ± 0.03	0.22 ± 0.03	0.26 ± 0.02	0.31 ± 0.04	0.32 ± 0.04	0.29 ± 0.03
C18:2 c9,t12	0.44 ± 0.04	0.42 ± 0.04	0.05 ± 0.01	0.05 ± 0.01	0.21 ± 0.02	0.18 ± 0.02	0.25 ±0.03	0.30 ± 0.03	0.41 ± 0.05	0.46 ± 0.04
C18:2 c9,c12	10.02 ± 1.01	9.48 ± 0.10	2.91 ± 0.18	2.78 ± 0.28	9.19 ± 0.10	8.57 ± 0.06	14.12 ±1.21	13.44 ± 1.18	11.08 ± 1.17	10.15 ± 1.01
C20:0	0.28 ± 0.03	0.33 ± 0.02	0.22 ± 0.04	0.28 ± 0.03	0.27 ± 0.02	0.32 ± 0.03	0.29 ± 0.03	0.33 ± 0.05	0.36 ± 0.04	0.31 ± 0.04
C20:1t	0.03 ± 0.00	0.03 ± 0.01	0.05 ± 0.01	0.06 ± 0.01	0.02 ± 0.00	0.02 ± 0.00	0.02 ± 0.00	0.02 ± 0.00	0.02 ± 0.00	0.03 ± 0.01
C20:1 c9	0.05 ± 0.01	0.06 ± 0.02	0.10 ± 0.02	0.09 ± 0.02	0.04 ± 0.01	0.05 ± 0.01	0.07 ± 0.01	0.09 ± 0.02	0.61 ± 0.05	0.07 ±0.02
C20:1 c11	0.41 ± 0.04	0.38 ± 0.03	0.41 ± 0.05	0.35 ± 0.05	0.29 ± 0.03	0.22 ± 0.03	0.17 ± 0.03	0.21 ± 0.03	0.39 ± 0.04	0.35 ± 0.05
C18:3 n3	0.18 ± 0.03	0.20 ± 0.02	0.51 ± 0.04	0.58 ± 0.03	0.17 ± 0.02	0.19 ± 0.03	0.39 ± 0.04	0.31 ± 0.03	0.18 ± 0.02	0.22 ± 0.06
C18:2 CLA	0.03 ± 0.01	0.03 ± 0.01	0.45 ± 0.04	0.42 ± 0.03	0.06 ± 0.01	0.08 ±0.01	0.02 ± 0.00	0.02 ± 0.00	0.02 ± 0.00	0.03 ± 0.01
C18:4	0.22 ± 0.02	0.25 ± 0.02	0.66 ± 0.06	0.55 ± 0.05	0.10 ± 0.01	0.11 ± 0.02	0.04 ± 0.01	0.05 ± 0.01	0.19 ± 0.02	0.24 ± 0.04
C20:3 n6	0.66 ± 0.07	0.58 ± 0.05	0.09 ± 0.02	0.08 ± 0.02	0.69 ± 0.02	0.52 ± 0.04	0.65 ± 0.03	0.59 ± 0.04	0.69 ± 0.06	0.62 ± 0.05
C20:4 n6 AA	0.19 ± 0.04	0.21 ± 0.02	0.25 ± 0.04	0.21 ± 0.02	0.08 ± 0.02	0.07 ± 0.02	0.02 ± 0.00	0.03 ± 0.00	0.09 ± 0.02	0.08 ± 0.02
C20:5 EPA	1.31 ± 0.08	1.25 ± 0.09	2.58 ± 0.12	2.47 ± 0.21	0.51 ± 0.01	0.57 ± 0.04	0.21 ± 0.02	0.19 ± 0.02	1.21 ± 0.14	1.28 ± 0.05
C22:5 DPA	0.12 ± 0.04	0.11 ± 0.08	0.39 ± 0.06	0.33 ± 0.04	0.04 ± 0.01	0.04 ± 0.01	0.02 ± 0.00	0.02 ± 0.00	0.14 ± 0.03	0.11 ± 0.02
C22:6 DHA	0.71 ± 0.06	0.67 ± 0.05	2.42 ± 0.24	2.31 ± 0.22	0.93 ± 0.03	0.87 ± 0.06	0.36 ± 0.03	0.32 ± 0.03	0.61 ± 0.05	0.52 ± 0.03
SFA	17.21 ± 1.25	16.59 ± 1.81	48.67 ± 3.40	48.17 ± 3.62	26.13 ± 2.01	25.66 ± 2.01	17.53 ± 1.31	16.17 ± 1.35	11.96 ± 1.10	11.17 ± 1.15
MUFA	69.99 ± 4.52	68.68 ± 4.95	37.82 ± 2.66	37.78 ± 2.91	62.55 ± 4.68	61.48 ± 5.64	69.03 ± 4.30	67.68 ± 5.95	76.22 ± 5.21	73.76 ± 6.12
PUFA	12.80 ± 1.05	13.46 ± 1.21	13.51 ± 1.10	9.89 ± 0.92	11.32 ± 1.04	11.43 ± 1.29	13.45 ± 1.21	15.58 ± 1.65	11.82 ± 103	14.01 ± 1.54
n6/n3		4.34 ± 0.07		0.53 ± 0.06		5.17 ± 0.64		16.16 ± 0.18		4.83 ± 0.54

FA: fatty acid; i: iso fatty acid; ai: anteiso fatty acid; c: *cis*; t: *trans*; AA: arachidonic acid; EPA: eicosapentanoic fatty acid; DPA: docosapentanoic fatty acids; DHA: docosahexanoic fatty acid; SFA: saturated fatty acid; MUFA: monounsaturated fatty acid; PUFA: polyunsaturated fatty acid; R: reception; shelf life (SL) 12 weeks (Mw1, Mw2, Mw3, Mw4 and Mw5) at room temperature; n: omega fatty acids.

**Table 4:** *Trans* monoenoic fatty acid isomers composition (mg FA/100 g of total FA ± Standard Deviation (SD) at the reception (R) and at the end of shelf life (SL) in commercial PUFA UHT-enriched milk.

tFA	Mw1		Mw2		Mw3		Mw4		Mw5	
	R	SL	R	SL	R	SL	R	SL	R	SL
Total *trans* 16:1	18.25 ± 1.41	19.15 ± 1.36	49.41 ± 4.01	48.75 ± 4.08	71.95 ± 6.95	69.80 ± 5.95	28.75 ± 2.61	30.95 ± 3.012	33.21 ± 2.84	35.60 ± 3.21
*trans* 4 C18:1	6.35 ± 0.08	6.44 ± 0.63	6.09 ± 0.52	6.18 ± 0.65	5.09 ± 0.51	5.97 ± 0.45	4.01 ± 0.41	4.29 ± 0.42	5.02 ± 0.45	4.16 ± 0.42
*trans* 5 C18:1	7.09 ± 0.07	7.68 ± 0.77	9.15 ± 0.62	9.47 ± 0.09	6.08 ± 0.08	5.81 ± 0.52	6.02 ± 0.52	6.65 ± 0.64	5.12 ± 0.51	5.31 ± 0.54
*trans* 6-8 C18:1	19.95 ± 1.64	16.39 ± 1.51 *	19.95 ± 1.38	18.85 ± 1.65	6.91 ± 0.05	9.07 ± 0.85	25.95 ± 2.36	19.84 ± 1.72	19.96 ± 1.84	14.19 ± 1.32 *
*trans* 9 C18:1	17.65 ± 0.58	18.11 ± 1.88	39.02 ± 3.08	40.12 ± 3.95	15.21 ± 1.21	13.93 ± 1.25	28.62 ± 2.95	30.12 ± 2.95	25.29 ± 2.04	19.07 ± 1.66 *
*trans* 10 C18:1	32.28 ± 0.31	36.19 ± 3.05 *	139.25 ± 11.35	123.04 ± 11.69	18.66 ± 1.32	17.23 ± 1.66	47.32 ± 3.74	45.16 ± 4.01	12.31 ± 1.18	13.18 ± 1.12
*trans* 11 C18:1	62.85 ± 5.41	64.61 ± 5.21	759.36 ± 62.04	761.79 ± 65.21	65.85 ± 5.65	62.72 ± 5.88	151.90 ± 14.13	145.06 ± 13.69	169.53 ± 17.02	157.90 ± 14.79
*trans* 12 C18:1	22.38 ± 2.38	28.23 ± 2.12	131.69 ± 11.24	137.64 ± 12.98	31.20 ± 3.12	32.80 ± 2.66	34.12 ± 2.74	36.74 ± 3.52	33.65 ± 3.12	31.27 ± 2.86
*trans* 13–14 C18:1	57.69 ± 5.01	59.43 ± 5.08	451.32 ± 32.54	442.42 ± 32.65	36.95 ± 3.8	33.94 ± 3.39	81.66 ± 7.62	85.87 ± 7.66	83.95 ± 7.96	78.80 ± 6.96
*trans* 15 C18:1	15.26 ± 1.62	16.48 ± 1.54	121.36 ± 11.02	132.18 ± 12.74	35.12 ± 0.29	33.48 ± 3.01	30.24 ± 3.01	31.29 ± 2.62	42.62 ± 3.29	38.49 ± 3.12
*trans* 16 C18:1	12.78 ± 1.02	13.98 ± 1.11	118.69 ± 10.95	127.02 ± 11.66	27.66 ± 0.21	29.72 ± 2.27	22.54 ± 2.12	25.10 ± 2.04	36.88 ± 3.08	32.82 ± 2.97

tFA: *trans* fatty acid; R: reception; shelf life (SL) 12 weeks (Mw1, Mw2, Mw3,Mw4 and Mw5) at room temperature; *: significance *p* < 0.05; *n* = 9.
